# Non-Cytotoxic Nanoparticles

**DOI:** 10.3390/nano12162865

**Published:** 2022-08-19

**Authors:** Raphaël Schneider

**Affiliations:** Université de Lorraine, CNRS, LRGP, F-54000 Nancy, France; raphael.schneider@univ-lorraine.fr

For several decades, nanoparticles (NPs) are becoming widely used in various industries, in electronics, agriculture, textile production or medicine. The benefits of using NPs are enormous, and NPs have found applications for many years in a wide variety of fields, among which we can mention as an example catalysis, electronic devices, removal of persistent pollutants from the environment, clothing, coating and sporting goods, diagnosing tools and treatment, cosmetics and personal care products, or food supplements [1,2,3].

The increase in the production of NPs and in their use inevitably results in their enhanced exposure to humans and the environment. Moreover, the marketing of NPs is faster than the time required to assess their risks on human health and the environment. For this reason, a significant effort is dedicated to research in the area of environmental and health safety of nanotechnology.

After entering the human body through inhalation, absorption, injection or ingestion, NPs may cross various cellular barriers and reach sensitive organs like the lung, the liver or the kidney. This may result in mitochondrial damage, DNA mutations and very often cell apoptosis [4,5]. The primary mechanism in NPs toxicity is the generation of reactive oxygen species (ROS) that may induce oxidative stress, which will have as consequences damages to cell membranes, to proteins, and to DNA [6]. It is also noteworthy that the level of ROS generated by NPs may depend on various parameters like their size, their morphology, their composition, their surface chemistry, their colloidal stability or their degree of agglomeration [7].

The environmental situation is also very complex and to assess the safety of NPs, it is crucial to master the release, the transport, the accumulation, the bio-transformation/degradation, and possibly the uptake of NPs by living beings that may be exposed [8].

At present, researchers as well as socio-economic actors often face the problem of balance between the positive effect of NPs and side effects related to their toxicity. Developing structure-activity relationships is needed to predict biological impacts, ecological impacts, and degradation at end-of-life. Each of these models is necessary to design NPs that will have the desired human health and environmental performance to complement their chemical and physical properties.

Some of these issues have been studied in this Special Issue, “Non-cytotoxic Nanoparticles”, and improvements have been proposed to reduce or eliminate any toxicity from NPs.

Studies on metal NPs or metal oxide NPs account for a large part of the items. In particular, (i) Ag nanocolloids allowing us to solubilize drugs with anticancer activity, (ii) Ag-based nanocomposites of low toxicity but exhibiting antibacterial activity, (iii) the use of taurine as a capping ligand for the preparation of stable and non-toxic Au NPs and (iv) an evaluation of the toxicity of metal (Ag, Ni) and metal oxide (Co, Cr) NPs have been developed. The cytotoxicity of various groups of inorganic NPs (ZnO, CuO, Fe_2_O_3_, SiO_2_,…) exhibiting different dispersibility in an aqueous solution was also investigated and further highlights that the toxicity is complex and dependent on many parameters. Two reviews are focused on a topical subject, the first one evaluated the toxicity of titanium-based materials, in particular contained in implants, and the second describes the influence of the TiO_2_ surface modification for fast osseointegration. The use of titanium peroxide NPs capped with polyacrylic acid exhibiting low toxicity and an excellent radiosensitizing effect is also described. Finally, a third review concerns an environmental problem and describes the adsorption, internalization, translocation and bioaccumulation by plants of C-, Ag-, Ce-, Ti-, Cu-, and Zn-containing engineered nanomaterials.

Another hot topic, carbon nanomaterials, was the subject of four publications. In recent years, carbon nanomaterials became valuable tools due to their unique properties (high conductivity, attractive optical properties, etc.).and have found applications in various areas including electronic devices, batteries, biology and medicine. A first study evaluated the toxicity of carbon nanomaterials (carbon nanohorns, multi-walled carbon nanotubes and carbon black), and a second one describes the preparation of biocompatible graphene layers. Two other studies are focused on graphene quantum dots (GQDs) and describe the use of these NPs as fluorescent probes for cell labeling as well as their antioxidant properties when adequately functionalized with diamino-PEG.

Finally, two articles are devoted to organic NPs. The first describes the use of temperature responsive submicrocapsules to protect and to decrease the toxicity of the Pyraclostrobin fungicide, and the second one the preparation of PEG-polyamine nanocomplex for the delivery of messenger RNA.

All the articles in this Special Issue demonstrate that understanding the mechanism of NPs toxicity will guide efforts to modify, redesign or develop new NPs that have small or no negative effects on health and/or environmental impact. These modifications and the preparation of new NPs will require us to confirm that the NPs still demonstrate the physicochemical properties that led to their integration into products or devices.

In conclusion, I would like to thank all of the authors who contributed to this Special Issue, as well as the members of the *Nanomaterials* editorial board for their joint work.
